# Psychiatry during the Nazi era: ethical lessons for the modern professional

**DOI:** 10.1186/1744-859X-6-8

**Published:** 2007-02-27

**Authors:** Rael D Strous

**Affiliations:** 1 Department of Psychiatry, Beer Yaakov Mental Health Center, Sackler Faculty of Medicine, Tel Aviv University, Tel Aviv, Israel

## Abstract

For the first time in history, psychiatrists during the Nazi era sought to systematically exterminate their patients. However, little has been published from this dark period analyzing what may be learned for clinical and research psychiatry. At each stage in the murderous process lay a series of unethical and heinous practices, with many psychiatrists demonstrating a profound commitment to the atrocities, playing central, pivotal roles critical to the success of Nazi policy. Several misconceptions led to this misconduct, including allowing philosophical constructs to define clinical practice, focusing exclusively on preventative medicine, allowing political pressures to influence practice, blurring the roles of clinicians and researchers, and falsely believing that good science and good ethics always co-exist. Psychiatry during this period provides a most horrifying example of how science may be perverted by external forces. It thus becomes crucial to include the Nazi era psychiatry experience in ethics training as an example of proper practice gone awry.

## Background

During the Nazi era, for the first time in history, psychiatrists sought to systematically exterminate their patients. It has been acknowledged that the medical profession was profoundly involved in crimes against humanity during this period, with various publications describing this malevolent period of medical history. It is less known, however, that psychiatrists were among the worst transgressors. At each stage of the descent of the profession into the depths of criminal and genocidal clinical practice lay a series of unethical decisions and immoral professional judgments. Furthermore, very little has been published on lessons that may be learned from this dark period in the history of psychiatry and on ethical principles that may be extrapolated for the future practice of clinical and research psychiatry and for inclusion in educational programs. This paper reviews the role of psychiatrists in the Nazi era and analyzes the underlying misconceptions that led to the aberrant behavior. Finally, some recommendations for inclusion of the study of this period in ethics training are presented [26].

## Role of psychiatrists in Nazi atrocities

The professional status of psychiatrists did not place any obstacle to their participation in Nazi crimes, and many demonstrated a profound commitment to the atrocities. Psychiatrists were instrumental in instituting a system of identifying, notifying, transporting, and killing hundreds of thousands of mentally ill and "racially and cognitively compromised" individuals in settings ranging from centralized psychiatric hospitals to prisons and death camps. Their role was *central *and *critical *to the success of Nazi policy, plans, and principles. Psychiatrists, along with many other physicians, facilitated the resolution of many of the regime's ideological and practical challenges, rather than taking a passive or even active stance of resistance [1]. Psychiatrists played a prominent and central role in two categories of the crimes against humanity, namely sterilization and euthanasia [2]. It was psychiatrists (many of whom were senior professors in academia) who sat on planning committees for both processes and who provided the theoretical backing for what transpired. It was psychiatrists who reported their patients to the authorities and coordinated their transfer from all over Germany to gas chambers situated on the premises of the six psychiatric institutions: Brandenburg, Grafeneck, Hartheim, Sonnenstein, Bernburg, and Hadamar [2,3]. It was psychiatrists who coordinated the "channeling" of patients on arrival into specially modified rooms where gassing took place. It was psychiatrists who saw to the killing of the patients (initially using carbon monoxide and later, starvation and injection). Finally, it was psychiatrists who faked causes of death on certificates sent to these patients' next of kin. It has been estimated that over 200,000 individuals with mental disorders of all subtypes were put to death in this manner [4-7]. Much of this process took place before the plan to annihilate the Jews, Gypsies and homosexuals of Europe. Hitler never gave the order to kill patients with mental illness. He only permitted it in a letter written in October 1939 and backdated to September 1, 1939 [2,6]. Psychiatrists were therefore never ordered to facilitate the process or carry out the murder of mentally ill...they were empowered to do so. Activity by psychiatrists and psychiatric institutions thus constituted the connection between euthanasia and the larger scale annihilation of Jews and other "undesirables" such as homosexuals in what came to be known as the Holocaust. Parenthetically, only one physician ever came to command an extermination camp. His name was Dr Imfried Eberl, a psychiatrist, who established Treblinka based on his experience as the Brandenburg Psychiatry Facility medical superintendent. He managed the camp for six months until he was fired for inefficiency in disposing of the thousands of bodies he succeeded in accumulating [2].

## Attitude of mainstream psychiatry to Nazi psychiatry practice following the war

While it would be expected that the involvement of psychiatrists in such a profound manner would be well-known in the field, this is not the case. Little has been published on the subject in mainstream psychiatry journals and even less is part of the formal education process for medical students and psychiatry residents. Several reasons may be proposed for this. First, it remains an embarrassment for the field that so many senior members – professors, department heads and internationally known figures – were so intimately involved. Second, many of those involved continued to practice and conduct research long after the war and were protected by colleagues. Third, and arguably most important, what psychiatrists did was based upon a paradigm shift in how patients and mental illness were viewed. Activities of psychiatrists became much of a value judgment in how they "read" the community and principles of neo-Darwinism with subsequent consideration of racial hygiene. In the absence of firm and unbending timeless ethical underpinnings to the practice of psychiatry, many felt that what they were doing was correct from a moral and scientific standpoint; therefore, they were not the demons and "paradigms of evil" that we perceive them to be. Their actions were a colossal misjudgment based on what today we may term "pseudoscience", but which at the time was deemed correct by many. Although actions based on "scientific theories" of mental illness in the past have led to patient deaths – one example being Henry Cotton and his belief that mental illness results from focal infection or chronic sepsis [8] – the extent and scale of the German psychiatrists' actions during the Nazi era remains unprecedented. These rationalizations based on faulty scientific theory and unethical medical practice were difficult to accept and therefore the nature and extent of these activities remained on the backbenches of the academic literature until more recently, when these issues have begun to be faced in an era of openness and transparency.

## Common assumptions leading to gross ethical misconduct

In addition to resting on poor science, the atrocities of the German psychiatric establishment were based upon several fundamental errors of ethical, professional, and scientific conduct. While many may simply brush off any deeper consideration of the issues with the stance that "they were just evil", such an approach only deepens the risk that such events will be repeated. The truth cannot be more different: perversion of ethical medical practice due to theoretical misjudgment and fundamental error in approach to the patient are what led to these atrocities of catastrophic proportions. So where did they go wrong? Several misconceptions lay at the source:

### 1) Medical ethics is ethnic, cultural, and time sensitive

The theory behind such a proposition is that much of medical ethics is time and culture bound [9]. Therefore what may be unethical now may not necessarily have been unethical then. This approach inculcates a relative attitude to the atrocities, minimizing the severity of the injustice and gross professional negligence so inherent in what transpired. Certain aspects of medical ethics transcend time and culture. Except under very specific and precise circumstances, such as when there is a serious and immediate risk to others, a physician should always respect autonomy, beneficence, and patients' confidentiality and dignity. Although it may be suggested that there is a major leap between disregard of these time-honored factors and the genocide of euthanasia, this is how it all began – it may even define the central thread of the atrocities to the mentally ill. While the form of ethical medical practice may depend on resources and cultural nuances (Tarasoff etc.), the basis for ethical behavior should remain constant, irrespective of time and place. Thus, while some maintain that for one generation a practice may be considered unethical but not for another [9], this is a misconception, as certain practices and concepts do not change with context. It is therefore never appropriate to kill one's patients *en masse *based on diagnosis and economic and racial-hygiene considerations for the community at large.

### 2) Philosophical constructs and ideas should define clinical practice

During the period of the Nazi regime, psychiatry supported compulsory sterilization and euthanasia of the physically and mentally ill, and subsequently, the killing of "inferior" races. They did this by applying scientifically invalid conclusions from evolutionary biology [10]. Aside from the fact that these philosophical constructs and scientific paradigms of evolutionary theory were flawed, they were also immoral and contravened basic tenets of medical ethics and clinical practice. Much of this approach was based on theories of neo-Darwinism. Furthermore, ever since Francis Galton in 1865 first published the idea of eugenics (a term rooted in the Greek "good in birth" or "noble in heredity"), individuals with mental illness had been targeted by eugenics programs, with psychiatrists intimately involved in the theoretical debate. The eugenics movement was not limited to Germany, and proponents of eugenics were prominent in several other countries including most notably Britain and the USA [11]. Interestingly, during the period in which euthanasia of the mentally ill was taking place in Germany, a fascinating debate transpired between two prominent American academics and was published in the *American Journal of Psychiatry *in 1942. Foster Kennedy, professor of neurology at Cornell University in New York, argued that all children with proven mental retardation ("feeblemindedness") over the age of five should be put to death. Leo Kanner, however, maintained that such individuals might still serve a purpose to society – garbage collection, postmen, etc. – as well as give meaning to their parents by virtue of having to care for them. Astoundingly, no one emphasized the unethical nature of putting individuals with disability to death. Instead, the editorial, published anonymously, appeared to side with Kennedy, and advised help for the parents in coming to terms with such a reality for their children and for the need for "enabling legislation" in order to facilitate the process legally (apparently in contrast to that of the German experience) [12]. The Nazi experience, which took much of the concept to fruition, was an extreme perversion of this movement, which existed already (at least at the conceptual level) in the minds of many psychiatrists supporting the idea.

### 3) Preventative medicine is more important than curative medicine

In the interests of preserving the future quality and purity of the Aryan race, racial hygiene became the battle cry of the German nation with Nazi medicine attempting to prevent the proliferation of illness. Within this context, it became the role of physicians in general, and psychiatrists in particular, to define who should be eliminated in order to best preserve the German nation's uniqueness and "higher-being". Thus in place of managing mental illness with the available tools (which were minimal) or investing resources in research for more appropriate treatment, it became important for physicians and psychiatrists to prevent such forms of illness or defects through euthanasia [7]. A particular focus was placed on psychiatric patients in the racial-hygiene program because they were perceived as weakening the "master-race" with no known cure. Therefore these "lives not worth living" were deemed useless and dangerous to German society and, in order to prevent their dissemination, the process of eliminating them in the context of the sterilization and euthanasia program came about. This procedure of trying to prevent illness, while a noble concept, should never be instituted at the expense of (and complete exclusion of) treating illness, as the disastrous Nazi program proved [7]. Even if one accepts their reasoning – and in this case they were wrong – selective sterilization of the mentally-ill would never significantly reduce the frequency of mental illness based on the Hardy-Weinberg law of preservation of rare recessive genes in a population of phenotypically normal carriers [10]. The Nazis embraced an exclusionary biological and racial determinism that removed any reparative function from clinical psychiatry. What remained was prevention of mental illness. Psychiatrists lived up to the challenge.

### 4) Psychiatrists have a particular role in channeling societal issues and public discussion

Many psychiatrists maintain that they have an inherent responsibility more than other medical professions to be involved in community affairs. This is because psychiatry by nature advocates a holistic approach to the patient, which often includes taking into account societal factors and contemporary ideology. Thus while the unique role of psychiatry in the genocide may be overstated, since other areas of medicine were also involved, psychiatrists fitted in particularly well. The dangers inherent in such involvement, while not obvious, are, however, prominent when important boundaries become blurred. Clinical practice and political machinations need to be kept separate. Many psychiatrists during the Nazi era were state-controlled and this further facilitated their conforming to the program. The rights of individuals cannot be totally ignored in the interests of society. The dangers become particularly acute in psychiatry compared to other subspecialties in medicine since it may be suggested that the field of psychiatry is often used in order to remove undesirables from society and place them in asylums. It may be argued that labeling of mental disease and its classification is a means of controlling members of the community who do not comply with accepted norms; therefore their freedom should be taken away and replaced with hospitalization. However, while at times there may be a fine line separating mental health and illness, it becomes very clear that the extent to which Nazi psychiatry allowed the political and community atmosphere to influence and govern clinical practice was grossly unethical, murderous, and unacceptable to an extreme extent.

### 5) Political and economic pressures may influence clinical practice

The management of patients must be dictated primarily by the patient's best interests and not by virtue of any ideology that may be prevalent at the time in society. This may include economic "ideological" considerations. Thus while pressures may exist "encouraging" the physician to make decisions one way or another based on the prevailing mood or tendency of the community at any time or place, this should be resisted and medical management should continue, unaffected by external considerations. The patient has to receive individual management and not be treated according to what is in vogue at the time. Psychiatrists should be wary of political and economic pressures that impinge upon medical decisions and health service provision. Nazism was supposed to be "applied biology" [13]. Science in general and psychiatry in particular needs to be independent from contemporary sociological and political contexts as well as protected from political abuse, even when embraced by the medical establishment. It has been proposed that the primary downfall of Nazi medicine was the failure of physicians to challenge the substantive core of Nazi values "Too many physicians were willing to go with the political flow; too many were unwilling to resist, to 'deviate' from 'commonly accepted' practices" [14]. Sound medical practice should be protected from the movement of political forces.

### 6) Psychiatrists/scientists have a responsibility to "enhance" mankind

Much of the early involvement by psychiatric clinicians and researchers in the process of "racial purification" arose from a genuine desire to improve mankind and not necessarily from the perspective of racist genocide. While no direct parallel can be drawn, today many continue in a sincere scientific effort towards the "enhancement" of man through molecular biology and genetic engineering [15]. Appropriate dialogue is required in order to ensure that the desire for "improving man", creating a "better human", does not come at the expense of the individual patient.

### 7) The interests of science take priority over the interests of the individual patient

Clinical management and research participation may appear to be equivalent, but they are not. A clear distinction must be made between the two and the patient must be aware of this. Research is critically important for the future of good medical practice and is fundamental to the philosophy of medical ethics in psychiatry which would be reflected in the long-term striving for excellence in clinical management. However, it should always be made clear to the patient that participation is voluntary and that more conventional treatment regimes exist and are available if preferred. Particular issues such as scientific validity, favorable risk-benefit ratio, voluntaryism, and decisional capacity, while important in all aspects of clinical practice, become of acute importance with respect to individuals with mental illness [16,17]. The Nazi experience, which completely disregarded such factors in the interests of "science" and racial-hygiene, is a prime example of the dangers inherent when such factors are not respected. Ethical commitment to research safeguards needs to be reflected in appropriate standards, guaranteeing appropriate study participation [16]. Refusal to participate in a study should likewise never interfere with the doctor-patient relationship and in the case of a patient agreeing to participate in research, it remains the duty of the physician to protect the health of the individual.

### 8) High-quality science and high-quality ethics always co-exist

It has become easy for those in the West to dismiss the depths of unethical medical practice of the Nazi physicians by categorizing it as bad science. This is easier to accept than the possibility that even within the context of good science, ethical behavior by physicians may go astray. In fact, the Nazi era in Germany was a time of remarkable scientific advances in several areas including cancer research and treatment, biochemistry, and quantum mechanics to name a few. In addition, the Nazis were pioneers of jet-propelled air flight, guided missiles, electronic computers, electron microscopes, and atomic fission [14]. Thus, scientific advancement does not necessarily go hand in hand with ethical advancement. It would be incorrect to brush off the ethical challenges that true scientific advancement in medicine may present, since the connection with true ethical practice is not necessarily a natural one.

## Ethics in the training of future psychiatrists

The theory of "medical ethics" has become a requirement in residency training programs in several countries around the world [18]. This has become a particularly pressing issue considering the need to understand principles of research ethics and roles of psychiatrists as investigators and researchers. The teaching of medical ethics during residency is particularly well-timed because professional identity and ethical practices are in their formative stages. Such ethics training is important in order to define the critical role that the four principles of autonomy, beneficence, non-maleficence, and justice play in clinical practice [19]. Several reports have been published that extol the virtues of medical ethics training in psychiatry residency training programs [20].

However, while the importance of such training programs is well recognized, so was the importance of medical ethics acknowledged in Germany in the 1930's. In fact, Germany possessed one of the most advanced and sophisticated codes of medical ethics in the world in existence from 1931. Some have even suggested that in certain aspects it was stricter than the subsequent Nuremberg Code or Helsinki Accord [14,21]. Doctors in general and psychiatrists in particular who were involved in the euthanasia program were not morally blind or devoid of the power of moral reflection. This belief would render the guilty parties not responsible for their actions. However, such codes did not help and when it came to bringing to fruition the ideology and plans of the wider society's beliefs, psychiatrists cooperated fully. They even took enthusiastic initiative in the process, allowing societal politics and ideas to interfere with clinical practice. Furthermore, although a broader consideration of potential abuse and malpractice in other totalitarian regimes would further strengthen the importance of the subject, a focus on Nazi psychiatric practice in particular brings to the fore clearly a most apt and recent example of how such interference can go awry. The example of Nazi psychiatry is a prime illustration of how ethics training without a focus on history is useless; where policy, even though existing, can be disregarded in the most grotesque fashion.

Furthermore, Cowley [19] has argued that although many medical schools have now given medical ethics a secure place in the curriculum, they err in treating it like a scientific body of knowledge. Ethics is a unique subject precisely because of its widespread relevance in all areas of life, and any teaching has to start from this shared understanding and from the familiar ethical concepts of ordinary language. Ethical jargon obscures the essential integration of ethics with the personal and "drives a wedge between ethical concepts and ethical conduct". This may have accounted for some of the unethical conduct of "Hitler's psychiatrists" in their disregard of basic principles despite the existence of strong ethical policy. "Ethical mantras" have little value when they exist away from a context of a mature understanding and self-reflection that needs to precede good ethical judgment and professionalism [19].

The question remains: why did so many psychiatrists willingly participate in the process of mass murder of the mentally ill? Perhaps some light may be shed on this issue by consideration of similar behavior as reported by Browning [22] in the history of an "ordinary" and unremarkable battalion of the Order Police that participated in mass shootings and deportations. Browning describes how these ordinary men were not coerced to kill, but rather participated in a willing fashion due to peer pressure, government sanction and following of orders, and in order to advance their own interests (careerism). This is in contrast to Goldhagen [23], who suggested that the average German citizen either participated in or ignored genocidal actions during the Nazi Era due to ingrained anti-Semitism, which was an intrinsic part of German society and had built up over centuries.

Yet another approach proposes that psychiatrists during the Nazi era were at particularly high risk for moral and ethical breaches because of how society and they themselves defined their role and power. Inherent in their work lay the risk of dehumanizing the patients with whom they had daily involvement, individuals at the extremes of human behavior. Moreover, psychiatry by nature incorporates contemporary ideology in its approach to the individual and society, and psychiatrists during that period were in essence state-controlled. All of these factors may have led to their tendency to objectify patients [reviewed in [1]]. Thus, these psychiatrists were primed to become involved in furthering Nazi ideology.

These differing approaches and considerations need to be conveyed to students of psychiatry, emphasizing that merely explaining the actions of psychiatrists and other physicians during this period by saying "they were evil" is misleading and reductionistic [24]. Danger exists in such an approach since it would preclude consideration of one's own risk for involvement in such a process.

Despite the wealth of ethics literature and the requirement for medical ethics in training programs, the experience of Nazi German psychiatry receives minimal mention, if at all, in contemporary medical student and resident ethics training courses. This is a serious oversight, since well-developed ethical principles did not stop the trespassing of political ideology into clinical practice and research in the 1930's. The result was equally devastating to the patients and to the practice of the profession. Every psychiatry resident needs to know about this. A knowledge and appreciation of Nazi psychiatry practices should become an important component of an integrated program of psychiatry ethics, with a focus on the human aspects of the psychiatric clinician and researcher, and a warning against the influences of political and community ideology impinging upon professional practice.

The content of ethics training for medical students and residents will remain a creative exercise for educators. Such a program should include an informative historical review of this period, including information on the unethical medical activities that transpired, case studies of psychiatrists at all levels of involvement, a consideration of various ethical frameworks for psychiatric care that were violated, as well as a consideration of why psychiatrists came to be so inextricably involved. Such a course would pave the way for a discussion about creating the most optimal framework of ethics for psychiatric practice. Several approaches may be considered, including the recent one by Bloch and Green [25], who proposed a model based on a "complementarity of principalism" (pragmatic focus on respect for autonomy, beneficence, non-maleficence and justice) and care ethics (highlighting character traits pertinent to caring for vulnerable psychiatric patients).

Thus, in addition to the usual formal medical ethics training in which the importance of autonomy, beneficence, confidentiality, and professionalism is emphasized, an in-depth appreciation of the actions of German psychiatrists during the Nazi years should be imparted. Since medical students and residents today are learning in an ethical environment that is unprecedented in its complexity [20], it becomes crucial to include the Nazi era medical experience as an example of proper practice gone awry, despite its being in the interest of science, and despite receiving the support of many of the foremost world leaders of the profession. The responsibility of psychiatrists to act as moral agents in the interests of patients, especially in the area of psychiatry, is thus of paramount value.

The professional burden of the memory of what transpired during the Nazi Era by the hands of members of the psychiatry profession is great. Those that were inextricably involved were colleagues, and this requires us to grapple with the intrinsic guilt of the profession, and to take responsibility to fix fundamental flaws in how we view patients and their management. A dark side to medicine exists: psychiatry, academia, and science played a key role in the establishment of National Socialism and all that ensued. The experience of psychiatry during the Nazi era provides an example of how science can be perverted by politics and therefore can become vulnerable to misuse and abuse. An exclusive focus on the monstrous aspects of Nazi medicine enables us to dismiss such events as aberrant and deviant, with a subsequent failure to internalize the inherent and very real dangers of the perversion of science and clinical management by outside political influences. Psychiatry cannot afford to turn a blind eye to such a past.

## Competing interests

The author declares that he has no competing interests.
